# Imaging for assessment of cancer treatment response to immune checkpoint inhibitors can be complementary in identifying hypophysitis

**DOI:** 10.3389/fendo.2023.1295865

**Published:** 2023-11-29

**Authors:** Anna Galligan, Amir Iravani, Arian Lasocki, Roslyn Wallace, Alison M. Weppler, Nirupa Sachithanandan, Cherie Chiang, Peter G. Colman, John Wentworth, Lavinia Spain, George Au-Yeung, Belinda Lee, Thomas W. H. Kay, Rodney J. Hicks, Shahneen Sandhu, Balasubramanian Krishnamurthy

**Affiliations:** ^1^ Immunology and Diabetes Unit, St Vincent’s Institute of Medical Research, Melbourne, VIC, Australia; ^2^ Department of Endocrinology and Diabetes, St Vincent’s Hospital, Melbourne, VIC, Australia; ^3^ Department of Medicine, The University of Melbourne, Melbourne, VIC, Australia; ^4^ Department of Cancer Imaging, Peter MacCallum Cancer Centre, Melbourne, VIC, Australia; ^5^ The Sir Peter MacCallum Department of Oncology, The University of Melbourne, Melbourne, VIC, Australia; ^6^ Department of Radiology, University of Washington, Seattle, WA, United States; ^7^ Department of Medical Oncology, Peter MacCallum Cancer Centre, Melbourne, VIC, Australia; ^8^ Department of Internal Medicine, Peter MacCallum Cancer Centre, Melbourne, VIC, Australia; ^9^ Department of Diabetes and Endocrinology, The Royal Melbourne Hospital, Melbourne, VIC, Australia

**Keywords:** hypophysitis, pituitary gland, immune related adverse events, combination immune checkpoint inhibition, immunotherapy, cancer imaging

## Abstract

**Introduction:**

Hypophysitis is reported in 8.5%–14% of patients receiving combination immune checkpoint inhibition (cICI) but can be a diagnostic challenge. This study aimed to assess the role of routine diagnostic imaging performed during therapeutic monitoring of combination anti-CTLA-4/anti-PD-1 treatment in the identification of hypophysitis and the relationship of imaging findings to clinical diagnostic criteria.

**Methods:**

This retrospective cohort study identified patients treated with cICI between January 2016 and January 2019 at a quaternary melanoma service. Medical records were reviewed to identify patients with a documented diagnosis of hypophysitis based on clinical criteria. Available structural brain imaging with magnetic resonance imaging (MRI) or computed tomography (CT) of the brain and 2-deoxy-2-[^18^F]fluoro-D-glucose positron emission tomography with computed tomography (FDG-PET/CT) were assessed retrospectively. The main radiological outcome measures were a relative change in pituitary size or FDG uptake temporally attributed to cICI.

**Results:**

There were 162 patients (median age 60 years, 30% female) included. A total of 100 and 134 had serial CT/MRI of the brain and FDG-PET/CT, respectively. There were 31 patients who had a documented diagnosis of hypophysitis and an additional 20 who had isolated pituitary imaging findings. The pituitary gland enlargement was mild, and the largest absolute gland size was 13 mm, with a relative increase of 7 mm from baseline. There were no cases of optic chiasm compression. Pituitary enlargement and increased FDG uptake were universally transient. High-dose glucocorticoid treatment for concurrent irAEs prevented assessment of the pituitary–adrenal axis in 90% of patients with isolated imaging findings.

**Conclusion:**

Careful review of changes in pituitary characteristics on imaging performed for assessment of therapeutic response to iICI may lead to increased identification and more prompt management of cICI-induced hypophysitis.

## Introduction

Spontaneous hypophysitis occurs in one in 7–9 million people per year (1). Widespread use of immune checkpoint inhibitors (ICI) against cytotoxic T-lymphocyte antigen 4 (anti-CTLA-4) and programmed cell death 1 (anti-PD-1) or its ligand (anti-PD-L1) for a range of advanced cancers in recent years has resulted in a dramatic increase in incidence (1–4). In the landmark CheckMate 067 trial evaluating combination ICI (cICI), the incidence of hypophysitis reported with ipilimumab and nivolumab combination, nivolumab, and ipilimumab was 7%, 1%, and 4%, respectively, suggesting that anti-CTLA-4 therapy is the major driver of this immune-related adverse event (irAE) (5, 6).

The European Society of Medical Oncology (ESMO) guidelines recommends regular monitoring of thyroid function in all patients treated with ICI, whereas pituitary hormone evaluation is generally reserved for clinical suspicion of hypophysitis (7). As symptomology can be non-specific or masked by glucocorticoid therapy for other concurrent irAEs, the true incidence of hypophysitis may be underestimated (2, 8).

At Peter MacCallum Cancer Centre, positron emission tomography with 2-deoxy-2-[^18^F]fluoro-D-glucose combined with computed tomography (FDG-PET/CT) and neuroimaging with either computed tomography (CT) or magnetic resonance imaging (MRI) are routinely used for the monitoring of melanoma patients (9) and for response assessment (10). The high glycolytic activity of activated T cells enables FDG-PET/CT to coincidentally detect immunotherapy-related inflammatory response in various tissues, including the pituitary (11). A recent study has reported MRI changes in the pituitary gland due to ICI-induced hypophysitis (12). Apart from small case series (10, 11, 13), the utility of FDG-PET/CT in detection of hypophysitis has not been systematically assessed. The complementary role of routine oncologic imaging for detecting irAEs, such as hypophysitis, in addition to monitoring the status of the patient’s metastatic disease, remains underappreciated.

This study aimed to assess the utility of routine diagnostic imaging in identifying cICI-induced hypophysitis with reference to clinical diagnoses based on symptoms and laboratory criteria.

## Methods

### Study design and participants

This retrospective cohort study at Peter MacCallum Cancer Centre was approved by the institutional ethics committee (approval number 17/231R). Pharmacy records were used to identify all patients who received at least one cycle of cICI for metastatic melanoma between January 2016 and January 2019 with a minimum of 6 months’ follow-up.

### Case identification

Patients receiving treatment with cICI for advanced melanoma have regular monitoring for safety and adverse events, including full blood count, liver function, electrolytes, and cortisol and thyroid function performed every 4–6 weeks. Patients may have additional cortisol levels and other pituitary hormones assessed in the setting of clinical or radiological suspicion. Patients who had biochemical evidence of secondary hypocortisolism in the absence of exogenous steroid use (8 a.m. cortisol < 150 or random cortisol <100 in the absence of concurrent glucocorticoid exposure) or secondary hypothyroidism or hypogonadotropic hypogonadism have been classified as documented hypophysitis. Patients who had imaging characteristics suspicious of hypophysitis without biochemical hormone deficiency were classified as having isolated pituitary imaging findings. As our pituitary imaging findings came from a blinded, retrospective review of pituitary appearance on brain imaging performed for the purpose of tumor response assessment, some patients with pituitary enlargement did not have biochemical testing as they never presented with symptoms of pituitary hormone deficiency. Many were receiving prolonged supraphysiological doses of glucocorticosteroids for treatment of concomitant irAEs, which may have masked symptoms of cortisol deficiency and would prevent accurate testing of the pituitary–adrenal axis.

### Pituitary imaging

FDG-PET/CT and neuroimaging with CT or MRI were undertaken as part of routine treatment response assessment, typically every 12–16 weeks during treatment unless expedited for clinical reasons. All studies were performed on an integrated PET/CT scanner including Biograph 16 (Siemens Medical Solutions, Erlangen, Germany) and GE 690 and GE 710 (GE Healthcare, Milwaukee, WI), with routine cross-calibration 3-monthly. FDG-PET/CT was performed as per European Association of Nuclear Medicine Research Ltd. (EARL) initiative (14).

### Variables and data measurement

Demographics, adverse events, and outcomes were collected retrospectively. In patients diagnosed with hypophysitis, symptomatology and available pituitary hormones were recorded. Cortisol deficiency was defined as a morning cortisol <250 nmol/L or random cortisol <150 nmol/L, and pituitary hormone parameters were interpreted according to ESMO guidelines (7).

In parallel, all available CT and MRI brain scans were assessed to determine whether the pituitary was adequately imaged. Evaluable MRI brain examinations included fine-slice pre- and post-contrast T1-weighted imaging in the axial plane, with multiplanar reconstructions. Sagittal reconstructions of the post-contrast T1-weighted sequence were used to measure gland size in the plane of the pituitary stalk. Pituitary size was assessed in the same plane on CT images. Pituitary size and appearance before and after treatment were retrospectively assessed by a neuroradiologist who was blinded to clinical information.

Imaging criteria for cICI-induced hypophysitis have not been established. We and others have previously published our experience of the key radiological characteristics as being modest, diffuse, and transient enlargement of the pituitary gland. A pituitary lesion seen at baseline or before treatment, heterogenous enhancement, or failure to resolve on serial imaging suggests alternate pathology (2, 15). For this study, we defined the criteria as (1) a relative increase in pituitary gland size by ≥3 mm (craniocaudally in the sagittal plane) after cICI or (2) in the absence of baseline imaging for comparison, an interval decrease in gland size of ≥3 mm on follow-up. In either case, the morphology and temporal evolution needed to also be consistent with hypophysitis, and not other differentials such as a pituitary metastasis. A difference in size of ≥3 mm was chosen as a confident indicator of a true change in size, whereas a smaller change could potentially be related to technical differences and/or observer variation. Equivocal changes were classified as negative (not affected) in the analysis. These criteria were based on the clinical experience of the reporting radiologist (15). CT and MRI changes were reviewed by an independent radiologist blinded to clinical information.

Serial FDG-PET/CT scans were assessed by two independent nuclear medicine physicians (NMPs) blinded to the clinical data. Majority agreement was used to define FDG-PET/CT detected hypophysitis. If only one user noted an increase in pituitary SUVmax, the result was considered equivocal. Equivocal results were classified as negative in the analysis. In the absence of established qualitative or semiquantitative criteria suggestive of hypophysitis on FDG PET/CT, for this study, a visibly perceptible increase in pituitary FDG uptake from baseline detected by both NMPs was considered positive. Using MIM software (MIM 6.7.11; MIM Software, Cleveland, OH), confirmatory semiquantitative analysis was performed with a 1-cm spherical region of interest over the pituitary fossa to assess maximum standardized uptake values (SUVmax) at baseline, post-treatment initiation, and until resolution. Thyroid and adrenal SUVmax were also assessed.

### Statistical methods

Baseline characteristics, prior treatment, and adverse event profiles in patients with and without hypophysitis were compared using Fisher’s exact, χ^2^ and Wilcoxon rank-sum tests. Interobserver agreement for the diagnosis of hypophysitis by FDG-PET/CT was assessed by the kappa statistic. Mann–Whitney U test and Kruskal–Wallis test were used for comparison of pituitary SUVmax and percentage increase in SUVmax, respectively, and were performed by GraphPad Prism 8 (GraphPad Software, La Jolla, USA).

## Results

### Participants

A total of 162 patients with a median age of 60 years (IQR 49–69) were included. There were 49 (30%) who had prior exposure to single-agent ICI, and 70 (43%) had received combination BRAF/MEK inhibition before cICI (**Table 1**).

**Table 1:** Clinical characteristics and immune toxicity.

	Clinical hypophysitis (n = 31)	Isolated radiological changes n = 20	Non-hypophysitis (n = 111)
No. (%) male	20 (64.5%)	12 (60%)	81 (73%)
No. (%) female	11 (35.5%)	8 (40%)	30 (27%)
Age, median (IQR), years	60 (46.5-68.7)	59.9 (54.4-69.7)	61.6 (48.6-68.9
No. (%) baseline ECOG			
0	27 (87.1%)	17 (85%)	79 (71.2%)
1	3 (9.7%)	2 (10%)	25 (22.5%)
2	2 (3.2%)	1 (5%)	6 (5.4%)
3	0 (0.0%)	0 (0%)	1 (0.9%)
No. (%) prior exposure to ICI^a^			
Ipilimumab	2 (6.5%)	2 (10%)	5 (4.5%)
Nivolumab	0 (0.0%)	0 (0%)	6 (5.4%)
Pembrolizumab	8 (25.8%)	5 (25%)	27 (24.3%)
No. (%) prior exposure to other treatment			
Chemotherapy	3 (9.7%)	0 (0%)	3 (2.7%)
BRAF/MEK inhibition	14 (45.2%)	10 (50%)	46 (41.4%)
No. (%) prior autoimmunity (where specified)			
≥1 autoimmune condition^b^	4 (12.9%)	2 (10%)	13 (11.7%)
Combination immune checkpoint inhibition (cICI)			
Total number of cycles, median (IQR)^c^	6 (3.0-15.0)	6 (3.0-11.25)	4.0 (2.0-11.0)
Immune-related adverse events			
No. (%) ≥1 irAE (other than hypophysitis)	27 (87.1%%)	19 (95%)	86 (77.5%)
No. (%) at least one grade 3/4 irAE	12 (38%)	12 (60%)	42 (37.8%)
No. (%) unplanned emergency or hospital presentation for irAE	24 (77%)	13 (65%)	47 (42.3%)
No. (%) organ-specific toxicity			
Dermatologic	15 (48.4%)	11 (55%)	39 (35.1%)
Thyroiditis	10 (32.3%)	9 (45%)	31 (27.9%)
Hepatitis	11 (35.5%)	8 (40%)	27 (24.3%)
Enteritis/colitis	12 (38.7%)	5 (25%)	30 (27.0%)
Rheumatic	4 (12.9%)	4 (20%)	11 (9.9%)
Pneumonitis	3 (9.7%)	4 (20%)	8 (7.2%)
Nephritis	2 (6.5%)	1 (5%)	1 (0.9%)
Myocarditis	0 (0%)	1(5%)	0 (0.0%)
Neurological	2 (6.5%)	0 (0%)	4 (3.6%)
Other^d^	2 (6.5%)	0 (0%)	2 (1.8%)

ECOG, Eastern Cooperative Oncology Group Performance Status; ICI, immune checkpoint inhibition.

There was no statistical difference between the groups for any characteristics.

a Some patients had two different single-agent treatments; for example, some patients initially had ipilimumab and then later pembrolizumab and have been included in both ipilimumab and pembrolizumab groups.

b Prior autoimmunity included rheumatoid arthritis, inflammatory bowel disease, psoriasis, vitiligo, Graves’ disease, Hashimoto’s disease, coeliac disease, and type 1 diabetes.

c Patients received four cycles of combination ipilimumab plus nivolumab followed by single-agent nivolumab.

d Other toxicities included lymphadenitis, hemolytic anemia, panniculitis-like T-cell lymphoma).

### Outcome data

**Figure 1:**
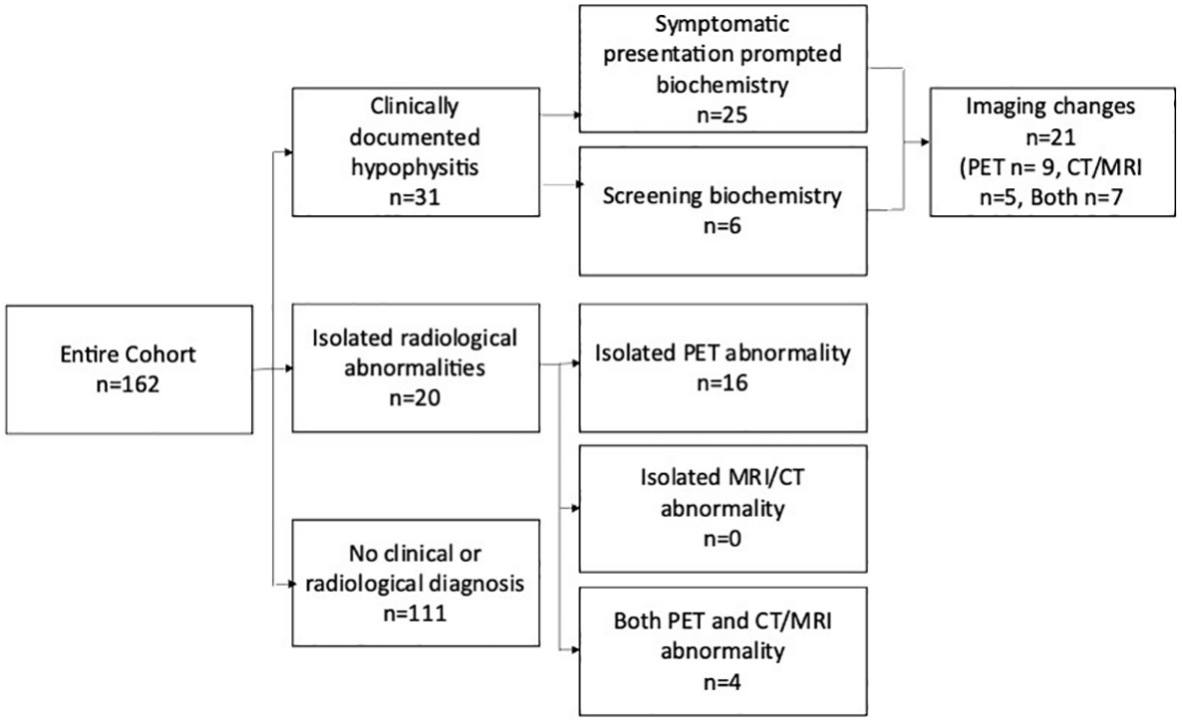
Schema highlighting the methods of detection of hypophysitis in the entire cohort. The 6 patients with clinically documented hypophysitis detected via screening biochemistry Includes three patients where abnormal imaging prompted biochemical screening. Patients with isolated radiological hypophysitis were detected retrospectively and did not have confirmatory pituitary hormones available. PET, positron emission tomography; CT, computed tomography; MRI, magnetic resonance imaging.

Clinical and biochemical evidence of hypophysitis was seen in 31/162 (19%), and an additional 20 (12%) had changes in pituitary images suggestive of hypophysitis (**Figure 1**). There was no difference in patient characteristics in patients with and without hypophysitis including prior treatment with single-agent anti-CTLA-4.

### Documented hypophysitis

In the 31 patients with a clinical diagnosis of hypophysitis, the median time to diagnosis was 9.6 weeks (range 0.7–40) after a median of 3 cycles (range 1–14). The majority (25/31) were diagnosed based on a low cortisol level after the patient reported new symptoms, most commonly lethargy (n = 18), headache (n = 16), or anorexia/nausea (n = 8). Visual disturbance was reported in two patients, and hypotension, weight loss, and delirium were single events. Asymptomatic screening or imaging-initiated cortisol and thyroid function tests led to the diagnosis in three and three patients, respectively (**Figure 1**). Pituitary hormone levels for the overall cohort are outlined in **Table 2** and **Supplementary Table 1**.

**Table 2:** Adrenal, thyroid, and pituitary function in the 31 patients with clinically documented hypophysitis.

**Adrenal Axis^a^**	Hypocortisolism		28/31
	Not assessed due to concurrent glucocorticoid requirement		3/31
**Thyroid Axis^b^**	Primary thyroiditis before hypophysitis onset (8/31)^c^	Recovery of primary thyroiditis	3/8
		Permanent secondary hypothyroidism	2/8
		Permanent primary hypothyroidism	3/8
	Primary thyroiditis at hypophysitis onset (3/31)^d^	Transient primary thyroiditis	1/3
		Permanent secondary hypothyroidism	1/3
		Permanent primary hypothyroidism	1/3
	No primary thyroid dysfunction (20/31)	Persistent euthyroidism	7/20
		Transient secondary hypothyroidism	5/20
		Permanent secondary hypothyroidism	4/20
		Not assessed	4/20
**Gonadal Axis**	Permanent hypogonadotropic hypogonadism		2/14
	Transient hypogonadotropic hypogonadism		5/14
	Not assessed		17/31

a A clinical diagnosis of hypophysitis was made by the clinician based on symptoms, anterior hormone levels, and, in some cases, pituitary imaging. In the three people receiving glucocorticoids, the diagnosis was made based on results of thyroid and gonadal function and imaging changes.

b To describe the evolution of thyroid function, patients were grouped according to those with prior thyroiditis, concomitant hypophysitis, and thyroiditis, and those with no evidence of thyroiditis.

c Eight patients had initially presented with transient thyrotoxicosis at a median of 100 days prior to hypophysitis onset. Three demonstrated permanent recovery of the thyroid gland, two evolved into secondary hypothyroidism, and three patients had persistent primary hypothyroidism. The persistently elevated TSH indicated an intact pituitary–thyroid axis.

d Two patients were clearly thyrotoxic at the time of acute cortisol deficiency, one demonstrated recovery of long-term thyroid function, and one evolved into permanent secondary hypothyroidism. One patient had primary hypothyroidism at the time of acute cortisol deficiency and at follow-up. The persistently elevated TSH indicated an intact pituitary–thyroid axis throughout the episode of hypophysitis.

**Figure 2:**
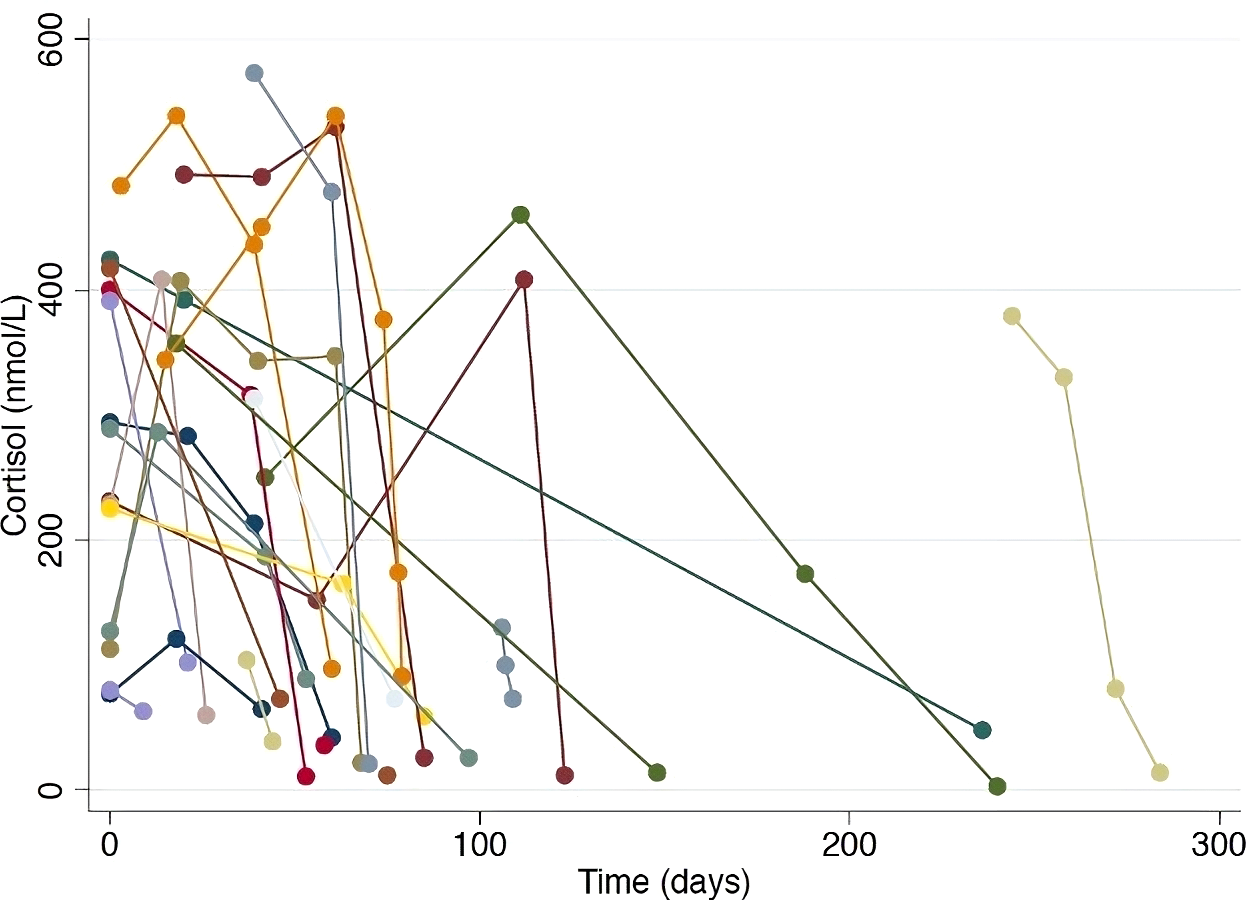
Serial cortisol levels in patients with documented hypophysitis. Each colored line represents an individual patient, demonstrating the precipitous drop in cortisol level observed in patients where regular cortisol testing was performed.

**Figure 2** outlines the precipitous drop from the normal range to a median nadir cortisol level of 42 nmol/L (range 3–102 nmol/L). Adrenocorticotropic hormone (ACTH) concentrations were measured in 18/31 patients, all of which were low, excluding primary adrenal insufficiency. Mild, asymptomatic hyponatremia occurred contemporaneously with cortisol deficiency in eight patients (**Supplementary Table 1**). In all clinically diagnosed cases, glucocorticoid replacement was commenced and subsequent withdrawal was not attempted.

### Thyroid dysfunction

When evaluating a patient with suspected hypophysitis, the following interpretations were made. Overt primary thyroid dysfunction is characterized by either a thyrotoxic state (high free T3 (fT3) and free T4 (fT4) with a suppressed TSH) or primary hypothyroidism (low fT3 and fT4 with an elevated TSH). Secondary hypothyroidism due to hypophysitis is characterized by low fT4 and low TSH. In the thyrotoxic state, the TSH will be suppressed and thyrotrope deficiency due to hypophysitis cannot be excluded until resolution of concurrent thyrotoxicosis. If the patient is taking thyroxine for primary hypothyroidism, pituitary–thyroid dysfunction will be masked and cannot be assessed.

In the 31 patients with a clinical diagnosis of hypophysitis, 11 patients had evidence of primary thyroid dysfunction (presenting with thyrotoxicosis in 10 and primary hypothyroidism in 1) preceding or at the time of cortisol deficiency (**Table 2**). Of these, thyroid function normalized in four patients, permanent secondary hypothyroidism developed in three patients, and in three patients permanent primary hypothyroidism evolved. The development of primary hypothyroidism as defined by a low T4 and high TSH in these three patients is a definitive indicator of an intact pituitary–thyroid axis. There were 20 patients with no evidence of primary thyroid dysfunction at hypophysitis onset. Nine of the 20 patients developed secondary hypothyroidism, which was transient in five patients and permanent in four. Thus, 22% (7/31) of patients diagnosed with hypophysitis developed permanent pituitary–thyroid axis dysfunction (3/7 with primary thyroiditis preceding secondary hypophysitis). Overall, long-term thyroxine replacement was required in seven (58%) of patients diagnosed with both thyroiditis and hypophysitis and four (20%) of patients with hypophysitis only (**Table 2**).

### Pituitary–gonadal axis

Pituitary–gonadal assessment including luteinizing hormone (LH), follicle-stimulating hormone (FSH), and testosterone (men)/estradiol (women) was available at hypophysitis diagnosis in 14 out of 31 patients, 7 of whom were hypogonadal. Persistent hypogonadotropic hypogonadism was observed in two of seven patients assessed at follow-up (**Table 2** and **Supplementary Table 1**). Diabetes insipidus did not occur. Prolactin and growth hormone were not routinely assessed.

### MRI/CT neuroimaging

MRI or CT imaging covering the pituitary fossa was available for evaluation in 100/162 patients. Baseline brain imaging was composed of MRI brain in 98 patients and CT brain in 17. Surveillance imaging of the brain with either MRI or CT generally occurred 2–4-monthly in patients with stage IV melanoma or more frequently if there were clinical symptoms warranting additional investigation (individualized based on the presence of brain metastasis at diagnosis). 73% performed within 2 months of commencing cICI, 23% within 4 months, and the remainder within 6 months. Reasons for a lack of posttreatment neuroimaging included short posttreatment survival and geographic/logistic limitations. Sagittal measurement of pituitary size was achievable in all scans. A change in pituitary size ≥3 mm occurred in 16/100 patients. In two of these, baseline imaging was not performed but sequential reduction in gland size by ≥3 mm was observed within 6 months of follow-up imaging. There were 12 out of these 16 patients who had a documented clinical diagnosis of hypophysitis and 11/16 patients who had an increase in pituitary uptake on FDG-PET/CT suggestive of hypophysitis (n = 11). The four patients without a documented diagnosis but with an increase in pituitary uptake on FDG PET/CT received long-term glucocorticoids for concomitant irAEs. Of these four patients, two died while on steroids, one patient remained on glucocorticoid for a concomitant irAE, and one patient had a normal cortisol after weaning the glucocorticoid.

**Figure 3:**
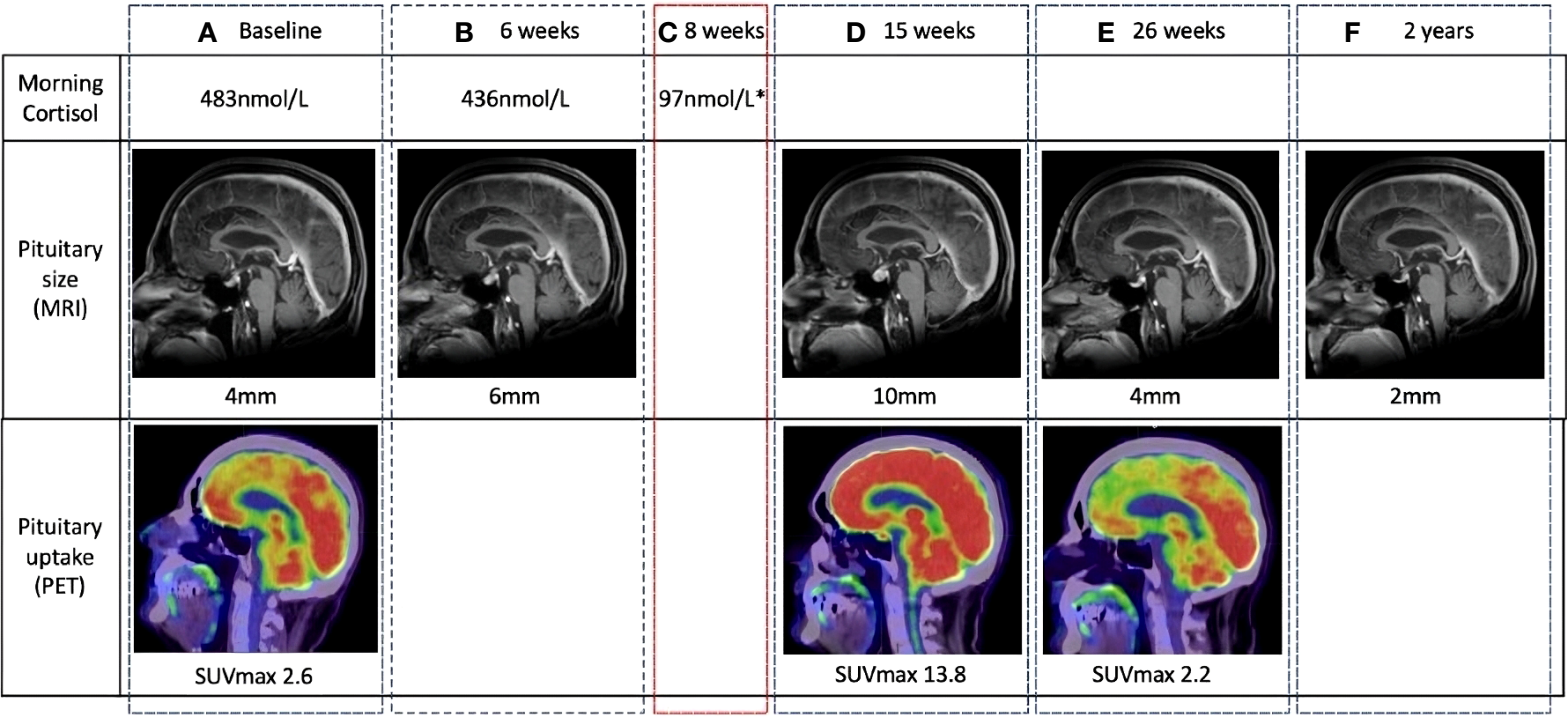
Temporal changes in pituitary gland size and metabolic activity in a patient with clinically diagnosed hypophysitis. The location of the pituitary fossa is indicated with a dotted white circle. **(A)** Normal baseline pituitary imaging and morning cortisol. **(B)** MRI performed at 6 weeks for surveillance of existing brain metastases demonstrates a 2-mm increase in pituitary size. Morning cortisol remains normal. **(C)** At 8 weeks, the patient complained of headache and lethargy. Morning cortisol was subnormal and a clinical diagnosis of hypophysitis was made. **(D)** 7 weeks after symptomatic cortisol deficiency, pituitary size, and metabolic activity are significantly increased from baseline on routine surveillance imaging. **(E, F)** Follow-up imaging demonstrates return to baseline pituitary size and avidity, and eventual reduction to sub-baseline size. MRI, magnetic resonance imaging (MRI); PET, positron emission tomography.

Typical evolution of pituitary size in a patient with documented hypophysitis is demonstrated in **Figure 3**. The largest absolute gland size was 13 mm, with a relative increase of 7 mm from baseline (normal pituitary size is 9 mm in women and 8 mm in men). There were no cases of optic chiasm compression. Imaging changes preceded a clinical diagnosis of hypophysitis in 6/16 patients, by a median of 18 days (range 5–82 days). Pituitary enlargement was universally transient. The mean final pituitary size was 5 mm and was not different between patients with and without a documented diagnosis of hypophysitis.

### Whole-body FDG-PET/CT

Serial FDG-PET/CT scans were undertaken in 134/162 patients. There were 42 patients who had available FDG-PET/CT imaging within 2 months from commencing cICI. A total of 81 patients had FDG-PET/CT within 3–4 months from commencing cICI, and the remaining had imaging 5–6 months from commencing cICI. The median time from commencement of cICI to the first PET scan was 76 days (range 18–225). A visibly perceptible increase in pituitary uptake suspicious for hypophysitis was noted by both observers in 36/134 patients at median 79 days from first treatment (range 20–126). Interobserver agreement between two NMPs was high, kappa 0.76 (95% confidence interval [CI] 0.64–0.88).

**Figure 4:**
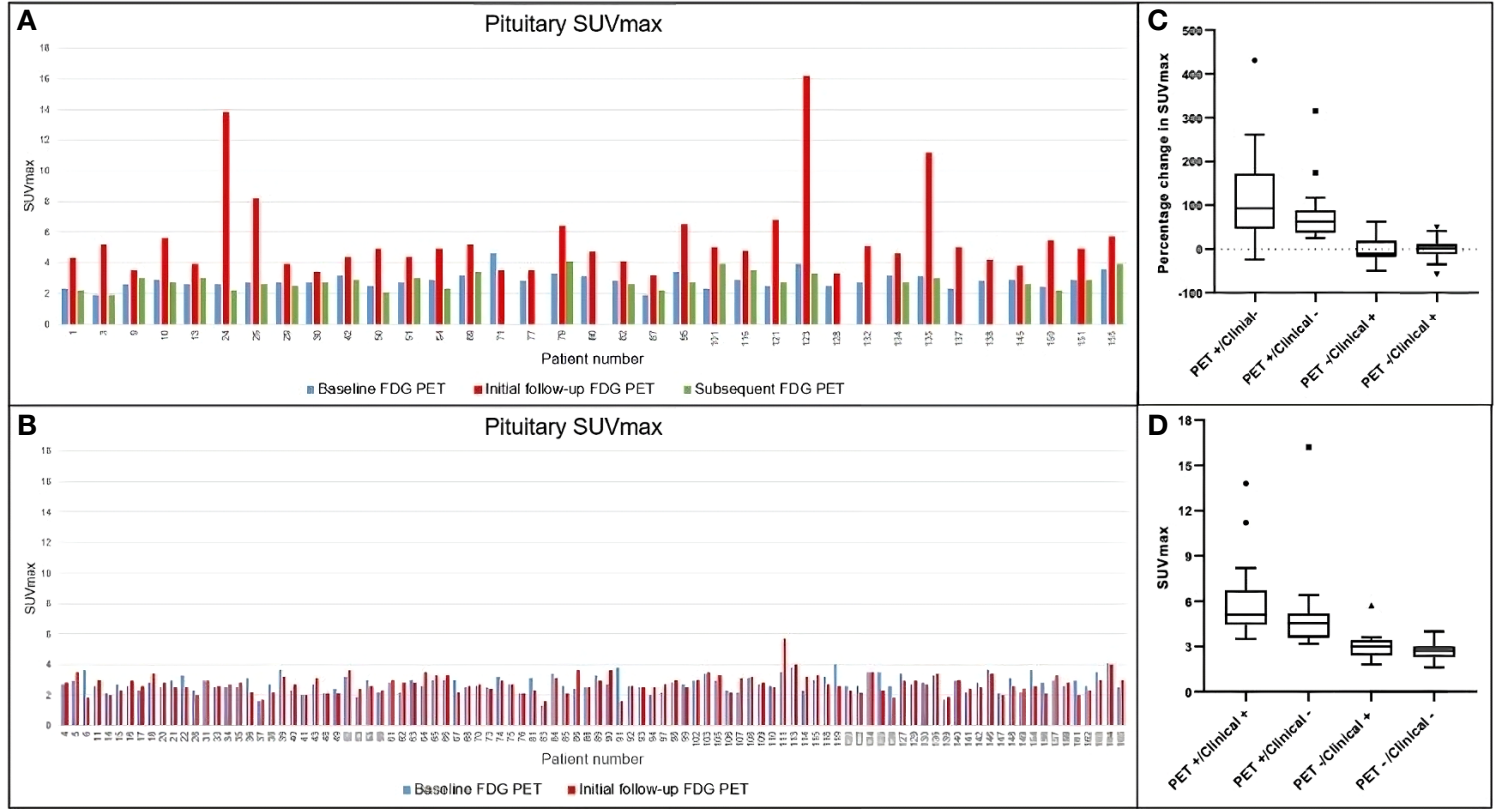
Temporal changes in pituitary SUVmax in the whole cohort. **(A)** Patients visually assessed as FDG PET-detected hypophysitis. Subsequent change in SUVmax is demonstrated in green bar if a further follow-up FDG PET was performed. **(B)** Patients without evidence of PET-detected hypophysitis. **(C, D)** Comparison of percentage change in SUVmax and absolute SUVmax among different groups. Both demonstrate significant difference in pituitary uptake among groups (p value for both <0.0001). SUVmax, maximum standardized uptake value; FDG PET, positron emission tomography with fluorodeoxyglucose.

**Figure 5:**
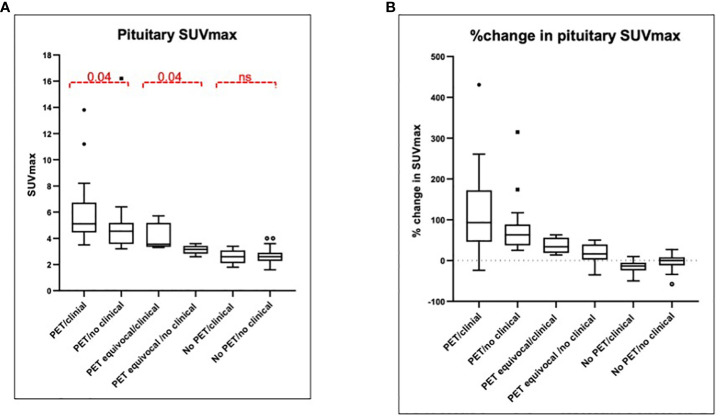
Pituitary SUVmax and percentage change at first follow-up. **(A)** Median SUVmax is presented among six groups depending on the PET findings: two users detect increased uptake (PET hypophysitis), one user detects increased uptake (PET equivocal) or both users agree with no increased uptake (no PET hypophysitis) and the presence or absence of a documented clinical diagnosis. The median SUVmax among the six groups was significantly different (p < 0.0001). **(B)** %change in pituitary SUVmax with respect to baseline is presented among the same six groups and was significantly different between the groups (p < 0.0001). SUVmax, maximum standardized uptake value; PET, positron emission tomography.

Typical evolution of pituitary SUVmax in FDG-PET/CT suggestive of hypophysitis is demonstrated in **Figure 3**. In the 36 patients with FDG-PET/CT suggestive of hypophysitis, the median SUVmax at diagnosis was 4.9 (IQR 3.9–5.6), with a corresponding increase of 68% (IQR 44–117) from baseline. Resolution of SUVmax to baseline metabolic activity occurred in all patients by 179 days (IQR 149–207) (**Figures 4**, **5**).

Of 31 patients with a documented diagnosis of hypophysitis, 16 (52%) had FDG-PET/CT changes, which preceded the clinical diagnosis in eight patients by a median of 22.5 days (range 5–182 days). Of the 20 patients with FDG-PET/CT changes without a clinical diagnosis, two had normal cortisol levels but no other pituitary hormones measured and 18 were receiving long-term glucocorticoids for another irAE. Of these 18 patients who were on glucocorticoids for another irAE, 10 died while on steroids, 6 weaned glucocorticoid, and 2 patients remained glucocorticoid dependent.

Of the 36 patients with FDG-PET/CT characteristics suggestive of hypophysitis, contemporaneous MRI was available in 31 and was corroborative in 11, equivocal in 6, and normal in 14 (**Supplementary Table 1**).

Overt increased uptake in the pituitary and thyroid gland was seen contemporaneously in 12 patients. Thyroid-stimulating hormone (TSH) was elevated, suggesting primary hypothyroidism at the time of increased pituitary SUVmax in seven patients. No increased uptake in the adrenal glands to suggest adrenalitis was observed.

## Discussion

Clinical and biochemical evidence of hypophysitis was found in 31/162 (19%), and additional 20/162 (12.3%) had pituitary imaging characteristics suggestive of hypophysitis in patients with advanced melanoma treated with cICI. The incidence of clinically documented hypophysitis in our patients was higher than the published incidence after cICI (19% versus 8%–14%) (5, 16, 17). Serial cortisol monitoring was not required in the CheckMate 067 trial, and our numbers likely reflect increased vigilance in monitoring for this toxicity. Importantly, a significant proportion of our cases were either detected or suspected on the basis of routine imaging. As such, temporal changes in pituitary size and FDG uptake on serial imaging may have a complementary role in facilitating the prompt diagnosis of hypophysitis.

Historically, autoimmune hypophysitis was a rare diagnosis usually made by exclusion of other causes of hypopituitarism or pituitary enlargement. With a higher index of suspicion after ICI, the accepted criteria for diagnosis relies on biochemical confirmation of anterior pituitary hormone deficiency in a symptomatic patient (7). This study and published reports suggest that the clinical syndrome of hypophysitis after ICI may be mild, non-specific, or masked by glucocorticoids.

While ESMO guidelines suggest measurement of LH, FSH, testosterone (men) and estradiol (women) in all suspected cases of hypophysitis, gonadotropic hormones may be unreliable due to acute or chronic illness suppressing the axis (18). We observed that assessment of the gonadal axis was infrequently undertaken in this cohort, which may have resulted in undetected hypogonadism. It is notable that the incidence of hypogonadism was 50% (7/14) when it was assessed, underpinning the need to evaluate the gonadal axis in the setting of immune-mediated hypopituitarism. Primary thyroiditis is common and must be considered when assessing the pituitary–thyroid axis. Thyroid hormone replacement after primary hypothyroidism may mask pituitary–thyroid dysfunction. Similarly, the pituitary–adrenal axis cannot be evaluated in the presence of supraphysiological doses of glucocorticoids (19). The majority of our cases detected exclusively by imaging had been prescribed glucocorticoids for concomitant irAEs, a common scenario during the first 6 months of treatment given the high incidence of irAEs from combination ipilimumab and nivolumab. Such pitfalls and an incomplete pituitary hormone panel in many patients were limitations of this retrospective study but also the case in the real world, making pituitary imaging highly complementary.

Traditionally, a dedicated pituitary MRI would be requested in a patient with suspected hypophysitis. While it is possible that subtle heterogeneity may be underappreciated without targeted pituitary imaging, we observed that the defining transient increase in pituitary size in hypophysitis can be detected on routine brain imaging performed for any indication. The temporal relationship between the size and appearance of the gland before and after ICI differentiates hypophysitis from alternate diagnoses. The two main findings—that (1) the absolute peak gland size at hypophysitis onset is often still within normal limits and (2) the enlargement is transient—suggest the diagnosis could be missed when the gland size is not compared with baseline or when there is a delay between hypophysitis onset and imaging. We, like others, have seen that radiological features of hypophysitis precede biochemical hypophysitis. The pituitary enlargement in ICI-induced hypophysitis is usually mild, and hence, in contrast to lymphocytic hypophysitis, visual defects are extremely rare (2, 20–23). Reports of increased pituitary FDG activity in patients with hypophysitis have raised the possibility of FDG-PET/CT, playing an important role in the diagnosis (11, 13, 15, 23).

We observed a transient increase in pituitary uptake in 36 patients with 16 having a confirmed clinical diagnosis. It is not known whether increased metabolic activity on FDG-PET reflects an early immune inflammatory response, or what effect early intervention with immunosuppression may have on reversibility of pituitary cell destruction. Only retrospective studies where glucocorticoids were administered after symptomatic cortisol deficiency are available and did not demonstrate a benefit (1). The findings of our study emphasize the added advantage of real-time assessment of pituitary size and FDG uptake in patients having longitudinal imaging for therapeutic monitoring of ICI therapy.

In our cohort, there were patients with a clinical diagnosis who did not have imaging changes. This may relate to the timing of the imaging. The biochemical diagnosis was made at a median of 9.6 weeks and the first CT/MRI or FDG-PET/CT was performed outside this window in some patients. The imaging in our study was performed for treatment response assessment at intervals determined by the treating clinician. As we have described modest and transient changes in the gland, there may have been patients where the window for identifying imaging changes was missed.

Moreover, imaging changes in ICI-related hypophysitis may be dependent on the type of checkpoint inhibitor. One study has demonstrated that pituitary enlargement was observed in nearly all patients with hypophysitis following treatment with ipilimumab, whether it was administered as monotherapy or in combination with nivolumab, but only in 30% of patients with hypophysitis induced by treatment with nivolumab or pembrolizumab alone (2).

An elevated TSH at the time of the abnormal FDG-PET/CT scan was a potential confounding factor in seven patients. Thyrotrope hyperplasia and pituitary hypermetabolism of FDG in the hypothyroid state are well described (24, 25). It is feasible that increased pituitary SUVmax may have been caused by physiological TSH overproduction in response to primary hypothyroidism in these patients.

Patients receiving treatment with immune checkpoint inhibitors undergo imaging before and after during treatment for assessment of response to treatment. Our findings’ careful review of pituitary gland on these radiological images for an increase in gland size or FDG uptake with respect to baseline might help in earlier diagnosis of hypophysitis. The pituitary imaging changes should more frequent monitoring of pituitary hormones. In a glucocorticoid-treated patient with isolated imaging changes, a paired morning cortisol and ACTH should be measured once the dosage is as low as 5 mg of prednisolone (or equivalent.) Patients should be referred to endocrinology for longitudinal evaluation, targeted hormone replacement, and evaluation for pituitary function recovery.

## Conclusions

Evaluation of routine structural assessment by MRI/CT and metabolic evaluation by FDG-PET/CT for specific changes in the pituitary gland may play a complementary role in facilitating the prompt diagnosis of hypophysitis.

## Data availability statement

The original contributions presented in the study are included in the article/**Supplementary Material**. Further inquiries can be directed to the corresponding authors.

## Ethics statement

The studies involving humans were approved by Peter Maccallum Cancer Centre Institutional Ethics Committee Approval number 17/231R (with waiver of patient consent). The studies were conducted in accordance with the local legislation and institutional requirements. The ethics committee/institutional review board waived the requirement of written informed consent for participation from the participants or the participants’ legal guardians/next of kin because of retrospective analysis of real world safety and efficacy of immunotherapy in patients with metastatic melanoma.

## Author contributions

AG: Conceptualization, Data curation, Formal analysis, Investigation, Methodology, Resources, Validation, Writing – original draft. AI: Conceptualization, Data curation, Formal analysis, Investigation, Methodology, Resources, Validation, Writing – original draft. AL: Investigation, Methodology, Writing – review & editing. RW: Investigation, Methodology, Writing – review & editing. AW: Investigation, Methodology, Writing – review & editing. NS: Investigation, Methodology, Resources, Writing – review & editing. CC: Investigation, Methodology, Resources, Validation, Writing – review & editing. PC: Resources, Supervision, Writing – review & editing. JW: Resources, Writing – review & editing. LS: Investigation, Methodology, Resources, Writing – review & editing. GA: Investigation, Methodology, Resources, Writing – review & editing. BL: Investigation, Methodology, Resources, Writing – review & editing. TK: Conceptualization, Formal analysis, Supervision, Writing – review & editing. RH: Investigation, Resources, Supervision, Writing – review & editing. SS: Conceptualization, Investigation, Project administration, Resources, Supervision, Writing – review & editing. BK: Conceptualization, Investigation, Supervision, Writing – review & editing.
