# Supplementary material for: Imaging for assessment of cancer treatment response to immune checkpoint inhibitors can be complementary in identifying hypophysitis
Source: Front Endocrinol (Lausanne). 2023 Nov 29;14:1295865. doi: 10.3389/fendo.2023.1295865 (PMC10716424; doi:10.3389/fendo.2023.1295865)
Supplement: Supplementary file 1 [file Table_1.docx]

**Supplemental Information**

This appendix has been provided by the authors to give readers additional information about their work.

Supplement to: Galligan, A, Iravani, A, Lasocki, A, Wallace, R, Weppler, A et al. The complementary role of routine cancer imaging in identifying clinically-occult hypophysitis after combination immune checkpoint inhibition.

**Supplemental Table 1:** Clinical, biochemical and imaging features of patients with a clinical or radiological diagnosis of hypophysitis.

|  | **Hypophysitis diagnosis** | **Symptoms^a^** | **Glucocorticoid use/indication** | **Pituitary hormones^b^** | **CT/MRI findings^c^** | **PET findings^d^** |
| --- | --- | --- | --- | --- | --- | --- |
| 1 | Isolated PET findings | None reported | Yes/  Pneumonitis, enteritis | Cortisol 58  ACTH 2.2 (while receiving glucocorticoid)  TSH 0.04, T4 10.5 (transient, recovered within 1 month  Other pituitary hormones not available | Normal MRI | PET hypophysitis |
| 2 | Isolated PET findings | None reported | Yes/  Thyroiditis, hepatitis, enteritis, pancreatitis | TSH 25.9, T4 <5.10  (pituitary-thyroid axis intact, required thyroxine)  Other pituitary hormones not available | Normal MRI | PET hypophysitis |
| 3 | Isolated equivocal MRI and equivocal PET findings | None reported | Yes/  Hepatitis | Random cortisol 248 after ceasing prednisolone  Other pituitary hormones not available | No increase in gland size but subjectively “bulky” appearance | Equivocal PET (1 user only) |
| 4 | Symptoms prompted biochemistry | Headache  Lethargy  Anorexia  Blurred vision | No | Adrenal and thyroid axes involved:  Cortisol 42 ACTH not available  Na 121  TSH 0.1, T4 8.2 | Normal MRI at time of biochemical diagnosis | Normal PET |
| 5 | Isolated PET findings | Abdominal pain at the time of low cortisol | Yes/  Nephritis | Cortisol 25 attributed to adrenal metastases, just prior to commencing glucocorticoids  Cortisol 365 2 months after cessation of glucocorticoids | Normal MRI | PET hypophysitis |
| 6 | Symptoms prompted biochemistry | Headache  Lethargy  Blurred vision | No  *note prior thyroiditis | Adrenal and thyroid axis involved:  Cortisol 12, ACTH <1.0, Na 137  TSH 0.32, T4 11.5 T3 1.9 LH 11.6, FSH 52.8, Oestrodiol 49 (appropriate post-menopausal range) | MRI hypophysitis | PET hypophysitis |
| 7 | Isolated PET findings Equivocal MRI findings | None reported | No | 1 out of 3 suboptimal random cortisol levels (237, 127, 320) in the 6 months post imaging  Transient changes suggestive of thyroid axis involvement  (TSH 0.14, T4 11.6)  Other pituitary hormones not available | No increase in gland size but subjectively “bulky” appearance and subsequent 2mm size reduction | PET hypophysitis |
| 8 | Isolated equivocal MRI changes | Hypotension | No | 1 out of 2 suboptimal morning cortisol levels (255, 172)  TSH 1.58 T4 13.1 T3 5.2  Other pituitary hormones not available | Equivocal MRI (1-2mm increase) | Normal PET |
| 9 | Isolated equivocal PET changes | None reported | No | Random cortisol levels 252, 200, 215  TSH 1.63 T4 11.2 T3 4.1 | Normal MRI | Equivocal PET (1 user only) |
| 10 | Isolated equivocal PET findings | Fatigue and postural hypotension while weaning glucocorticoid | Yes/  Colitis  *note prior thyroiditis | ACTH 3.5, cortisol 191 (on glucocorticoids) TSH 1.02, T4 10.0, T3 3.5  Random cortisol 179 after glucocorticoids ceased  Other pituitary hormones not available | Normal MRI | Equivocal PET (1 user only) |
| 11 | Symptoms prompted biochemistry | Lethargy | No | Adrenal axis only:  Cortisol 12, ACTH 1.9, Na 136  On thyroxine at presentation, euthyroid.  LH/FSH normal | MRI not available | Normal PET |
| 12 | Symptoms prompted biochemistry | Headache  Lethargy | No | Adrenal and thyroid axes involved:  Cortisol 97,  ACTH not available,  Na 140  TSH 3.5, T4 6 (required thyroxine)  Normal testosterone at follow up | MRI hypophysitis | PET hypophysitis |
| 13 | Symptoms prompted biochemistry | Headache | No | Adrenal, thyroid and gonadal axes involved: Cortisol 89 ACTH <3 Na 135  TSH 1.43 T4 <5.10 Testosterone 0.8 | MRI hypophysitis | PET hypophysitis |
| 14 | Symptoms prompted biochemistry | Headache  Lethargy | No | Adrenal and thyroid axes involved: Cortisol 11, ACTH 1.8, Na 136  TSH 0.09, T4 7.7 Testosterone 25.3 2 months later | MRI hypophysitis | FDG-PET not available |
| 15 | Isolated MRI and PET findings | None reported | Yes/  Hepatitis | Cortisol 286 (on glucocorticoid)  Transient evidence secondary hypothyroidism (TSH 0.19 T4 9.60)  Other pituitary hormones not available | MRI hypophysitis | PET hypophysitis |
| 16 | Isolated PET findings | None reported | Yes/  Arthritis, sicca syndrome | Random cortisol 132 while on prednisolone 10mg  TSH 0.78 at the time of PET, and persistently normal after  Other pituitary hormones not available | MRI not available | PET hypophysitis |
| 17 | Low cortisol on safety bloods | Lethargy | Yes/  Colitis | Adrenal axis only**:**  Cortisol 102, Na 143 (just before commencing glucocorticoids)  Other pituitary hormones not available at diagnosis  Normal TFTs at follow up (TSH 0.88, T4 13.8) | Normal MRI | PET hypophysitis |
| 18 | Symptoms prompted biochemistry | Lethargy | No | Not available (managed externally) | MRI not available | Normal PET |
| 19 | Symptoms prompted biochemistry | Lethargy | Yes/  Pneumonitis | Adrenal axis only**:**  Cortisol 12 after steroids ceased. ACTH not available  Primary thyroiditis treated with thyroxine  Normal gonadotropes at follow up | Normal MRI | PET hypophysitis |
| 20 | Isolated PET findings | None reported | Yes/  Pneumonitis, thyroiditis | TSH 31.3, T4 9.7, T3 2.5 (pituitary thyroid axis intact)  Other pituitary hormones not available | Normal MRI | PET hypophysitis |
| 21 | Isolated PET findings  Later unable to be weaned from steroids due to low cortisol | None reported | Yes/  Arthritis | Cortisol 62, TSH 1.29 T4 11 at the time of abnormal PET (was receiving prednisolone).  No gonadotropes measured.  6 months later after ceasing prednisolone, cortisol 101 and ACTH <0.3, attributed to adrenal axis suppression.  TFT remained normal (TSH 2.05 T4 12.7) | Equivocal MRI (1-2mm increase) | PET hypophysitis |
| 22 | Low cortisol on safety bloods | Lethargy  Dehydration | No  *note prior thyroiditis | Adrenal axis only:  Cortisol levels 130, 100, 73 ACTH not available,  Na 141  Transient primary thyroiditis (TSH <0.01 T4 37.4), no gonadotropes | Normal MRI | Normal PET |
| 23 | Abnormal MRI reported to clinicians, diagnosis made without biochemistry | Headache | Yes/  Colitis | Na 134  TSH 0.67 and remained normal  Other pituitary hormones not available | MRI hypophysitis | Equivocal PET (1 user only) |
| 24 | Symptoms prompted biochemistry | Lethargy | No | Adrenal axis only:  Cortisol 136, then <20  Na 136  ACTH 2.1, TSH 8.93 (intact pituitary thyroid axis), LH/FSH normal no testosterone | MRI not available | Normal PET |
| 25 | Isolated equivocal PET changes | None reported | No | Morning cortisol 301 1 month prior to abnormal PET  Transient secondary hypothyroidism  (TSH 0.39 T4 8.9) | MRI not available | Equivocal PET (1 user only) |
| 26 | Isolated PET findings | None reported | Yes/  CNS metastases,  Thyroiditis, rash | Random cortisol 183-366 in between glucocorticoid courses  TSH 9.34, T4 6.2 (Pituitary thyroid axis intact) | Normal MRI | PET hypophysitis |
| 27 | Symptoms prompted biochemistry | Lethargy  Anorexia | No | Adrenal axis only:  Cortisol 22 ACTH <1.0, Na 133  TSH 0.10, T4 14.9  6 months later developed primary hypothyroidism (TSH 14.4, T4 9.7) indicating intact pituitary thyroid axis  LH/FSH low at follow up | MRI hypophysitis (decrease in gland size >3mm) | PET hypophysitis |
| 28 | Abnormal PET prompted biochemistry | None reported | Yes/  Colitis | Adrenal, thyroid and gonadal axes:  On steroids at diagnosis, unable to be weaned. Secondary hypothyroidism (TSH 0.42 T3 2.3 T4 10.7) low LH and FSH | Equivocal MRI (decrease in gland size 2mm) | PET hypophysitis |
| 29 | Isolated PET findings | None reported | Yes/  Hepatitis | At the time of abnormal PET, TSH 0.04, T4 12.7 T3 3.1  After cessation of glucocorticoid: cortisol 332, ACTH 7 TSH 1.13 T4 13.9 | Normal MRI | PET hypophysitis |
| 30 | Isolated MRI and PET findings  Later unable to be weaned from steroids due to low cortisol | None reported | Yes/  Hepatitis | Pre-dose cortisol 21 while on 5mg prednisolone  TSH 1.99 T4 6.7 | MRI hypophysitis | PET hypophysitis |
| 31 | Isolated PET findings | None reported | Yes/  CNS disease | Cortisol 15 (recently prescribed dexamethasone, unclear if taking at the time of the test)  TSH 1.17, T3 2.1, T4 11  (Suspicious for thyroid axis involvement) | Normal MRI | PET hypophysitis |
| 32 | Symptoms prompted biochemistry | Headache | No | Adrenal, thyroid and gonadal axes:  Cortisol 60, ACTH 22,  TSH 0.86, T4 10.6  low LH | MRI not available | PET hypophysitis |
| 33 | Isolated equivocal PET changes | None reported | Yes/  Arthritis | At the time of the abnormal PET, cortisol 47 (on prednisolone)  TSH 0.68, T4 12.5  After prednisolone ceased, Morning cortisol 561  TFT remained normal | MRI not available | Equivocal PET (1 user only) |
| 34 | Isolated PET findings | None reported | No | At the time of abnormal PET, morning cortisol 209, TSH 3.0  TFT and cortisol normal 2 months later.  Other pituitary hormones not available | Normal MRI | PET hypophysitis |
| 35 | Symptoms prompted biochemistry | Lethargy | No | Adrenal and thyroid axes: Cortisol 59 ACTH not available, Na 136  TSH 1.18, T4 8.80 T3 1.9 | MRI images inadequate but pituitary appears to decrease in size | Equivocal PET (1 user only) |
| 36 | Isolated equivocal PET changes | Lethargy | No | Cortisol 604, 334  TSH 1.63 T4 11  Other pituitary hormones not available | MRI not available | Equivocal PET (1 user only) |
| 37 | Symptoms prompted biochemistry | Lethargy  Nausea | No | Adrenal axis only:  Cortisol 14, ACTH not available,  Na 130  TSH 0.84, T4 9.50 (persistent euthyroidism) | MRI not available | PET hypophysitis |
| 38 | Isolated PET findings  Later unable to be weaned from steroids due to low cortisol | None reported | Yes/  CNS disease, thyroiditis, rash/SJS | Pre-dose cortisol level 13 while on 5mg prednisolone  TSH 68, T4 5 (pituitary thyroid axis intact) | Normal MRI | PET hypophysitis |
| 39 | Isolated equivocal PET changes | None reported | No | Cortisol 536, TSH 2.24 T4 10.7 3 2.3 | Normal MRI | Equivocal PET (1 user only) |
| 40 | Symptoms prompted biochemistry | Delerium | No | Adrenal, thyroid and gonadal axes:  Cortisol 73, ACTH not available  TSH 0.22, T4 8.6  LH/FSH low | MRI not available | Equivocal PET (1 user only) |
| 41 | Isolated equivocal PET changes | None reported | No | Random cortisol 217, TSH 0.5 T4 14.8 | Normal MRI | Equivocal PET (1 user only) |
| 42 | Isolated equivocal MRI | Neurological changes attributed to CNS disease | Yes/  CNS disease, hepatitis | TSH 0.08 T4 12.1 T3 3.1  Other pituitary hormones not available | Small increase in gland size with “bulky” appearance | Normal PET |
| 43 | Isolated equivocal MRI before cICI (while taking PD-1 monotherapy) | None reported | Yes/ Arthritis | Hyponatraemia  Cortisol 40, unclear if on glucocorticoids | Small increase in gland size with “bulky” appearance | Normal PET 3 months after abnormal MRI |
| 44 | Low cortisol on safety bloods | Lethargy  Anorexia | No  *Prior thyroiditis | Adrenal axis only:  Cortisol 65 ACTH 0.6, Na 135  TSH 0.01 T4 14 (recovering thyroiditis)  High TSH at follow up indicates intact pituitary thyroid axis. LH/FSH normal at follow up. | Normal MRI 2 months after presentation | Equivocal PET (1 user only) |
| 45 | Isolated equivocal PET changes  Later unable to be weaned from steroids due to low cortisol | Headache  (attributed to CNS disease) | Yes/  CNS disease, colitis, thyroiditis, encephalomyelitis | Cortisol 19 after ceasing steroids ACTH not available  TSH 35, T4 2.6 (intact pituitary thyroid axis)  Gonadotropes not available | Normal MRI | Equivocal PET (1 user only) |
| 46 | Isolated PET findings | Headache and nausea  (attributed to CNS disease) | Yes/  CNS disease, pneumonitis, thyroiditis | Cortisol 125 after ceasing glucocorticoids  TFT normal at time of abnormal PET. A month later, transient secondary hypothyroidism TSH 0.26 T4 8.6 | Normal MRI | PET hypophysitis |
| 47 | Isolated equivocal PET changes | None reported | No | Cortisol 448, no ACTH  TSH 55 T4 <5.1 (intact pituitary-thyroid axis)  Gonadotropes not available | Normal MRI | Equivocal PET (1 user only) |
| 48 | Symptoms prompted biochemistry | Headache | No | Adrenal axis only:  Cortisol levels 73 and 26, ACTH 5.2  Na 142  TSH 0.51, T4 11, T3 3.4  LH/FSH normal Euthyroid at follow up | No increase in gland size but subjectively “bulky” appearance | PET hypophysitis |
| 49 | Isolated MRI and PET findings | Headache  (attributed to CNS disease) | Yes/  Hepatitis | One undetectable cortisol (<14) just prior to starting glucocorticoids  TSH 35, T4 4 (Primary hypothyroidism) at the time of abnormal scan | MRI hypophysitis | PET hypophysitis |
| 50 | Isolated PET findings | None reported | Yes/  Colitis | Cortisol 223, ACTH 2.3 (on prednisolone)  TSH 0.3, T4 12.10  (not repeated) | Normal MRI | PET hypophysitis |
| 51 | Isolated PET findings | None reported | Yes/  CNS disease, nephritis | None available | Normal MRI | PET hypophysitis |
| 52 | Symptoms prompted biochemistry | Headache  Malaise | No | Adrenal and gonadal axes: Cortisol 14 ACTH<1.0,  Na 119  TSH 0.68 T4 15.3 FSH 3.9 Testosterone 7.1 | No increase in gland size but subjectively “bulky” appearance | PET hypophysitis |
| 53 | Abnormal PET and MRI prompted biochemistry | Lethargy  Anorexia | No | Adrenal, thyroid and gonadal axes:  Cortisol 91, ACTH not available,  Na 136  TSH 0.3, T4 8.7 Hypogonadism at follow up. | MRI hypophysitis | PET hypophysitis |
| 54 | Symptoms prompted biochemistry | Headache  Dizziness | Yes  Vitiligo, pancreatitis, colitis | Not available (offsite) | MRI hypophysitis | PET hypophysitis |
| 55 | Low cortisol on safety bloods | None reported | No | Adrenal axis only:  Cortisol 26 no ACTH, Na 142  TSH 2.28 T4 10.7 | MRI not available | PET hypophysitis |
| 56 | Symptoms prompted biochemistry | Lethargy | No | Adrenal and gonadal axes: Cortisol 22 ACTH 4.5, Na 130, testosterone 4.2 TSH 8.85, T4 <5.10  transient low testosterone, permanent primary hypothyroidism required thyroxine | MRI hypophysitis | Normal PET |
| 57 | Isolated abnormal MRI and PET  Later unable to be weaned from steroids due to low cortisol | None reported | Yes  Pneumonitis, thyroiditis | Pre-dose cortisol 14 while on prednisolone 10mg.    TSH 5.53, T4 <5.10 (intact pituitary thyroid axis), Appropriate LH and FSH for post-menopausal state | MRI hypophysitis | PET hypophysitis |
| 58 | Symptoms prompted biochemistry | Lethargy | Ceased a month prior to diagnosis | Adrenal axis only:  Cortisol 63 ACTH 5.8 Na 133  Other pituitary hormones not available | MRI not available | Normal PET |
| 59 | Symptoms prompted biochemistry | Headache | No | Adrenal and thyroid axes:  Cortisol 39, ACTH <0.7, TSH 0.10 T4 15.8 (transient secondary hypothyroidism) Normal LH/FSH  Subsequent high TSH indicated recovery of pituitary thyroid axis | Normal MRI | Normal PET |
| 60 | Symptoms prompted biochemistry | Headache  Nausea | No | Adrenal axis only:  Cortisol 73 ACTH 4.5  TSH 0.49 T4 14.2, T3 4.5 (Transient low TSH) no gonadotrophes  Normal testosterone (23.9) at follow up | No increase in gland size but subjectively “bulky” appearance | PET hypophysitis |
| 61 | Symptoms prompted biochemistry | Headache | No | Adrenal, thyroid and gonadal axes:  Cortisol 21, no ACTH, Na 132  TSH 0.2, T4 11.60  Low FSH and LH for menopausal state | MRI hypophysitis | PET hypophysitis |
| 62 | Isolated PET findings | None reported | Yes,  CNS disease, colitis, thyroiditis | Cortisol 204 just prior to commencing glucocorticoids  TSH 30, T4 6 (intact pituitary thyroid axis) | MRI not available | PET hypophysitis |
| 63 | Safety bloods demonstrated secondary hypothyroidism | None reported | Yes/  Colitis, hepatitis | Thyroid axis only: TSH 0.2 T4 10.3 T3 3.8 **(**Permanent secondary hypothyroidism) | MRI hypophysitis | Normal PET 3 weeks before presentation |
| 64 | Symptoms prompted confirmatory biochemistry | Headache | Yes/  Hepatitis, autoimmune haemolytic anaemia | Adrenal axis only:  Cortisol 114 just prior to starting glucocorticoids Na 142  Other pituitary hormones not available  Euthyroid at follow up (TSH 2.02, T4 12) | MRI hypophysitis | Normal PET |

Table legend:

Abbreviations: Computed Tomography (CT), Magnetic Resonance Imaging (MRI), Positron Emission Tomography (PET), adrenocorticotripic hormone (ACTH), Sodium (Na), thyroid stimulating hormone (TSH), tetraiodothyronine (T4), triiodothyronine (T3), leutenizing hormone (LH), follicle stimulating hormone (FSH)

^a^ Symptoms attributed to hypophysitis or reported at the time of abnormal imaging and not explained by a known alternate cause

^b^ Pituitary hormones and biochemistry units and reference ranges: Cortisol nmol/L (morning >250, random >200), ACTH ng/L (7.2-63.3), Na mmol/L (135-145), TSH mU/L (0.35-4.94), T4 pmol/L (9.0-19), T3 pmol/L (2.6-5.7), Testosterone (males) nmol/L (8.3-30.2), LH and FSH are described as low, high or appropriate as adjudicated by an endocrinologist, based on the sex, age and gonadal status

^c^ CT/MRI criterion for hypophysitis was a change in pituitary size >/= 3mm after combination immune checkpoint inhibition. Features including subjective bulkiness or a change in pituitary size <3mm are illustrated in this table as equivocal and were classified as negative (no MRI detected hypophysitis) in the analysis

^d^ Majority agreement was used to define PET hypophysitis. If only one user detected an increase in pituitary uptake, the findings are illustrated as equivocal in this table and classified as negative (no PET detected hypophysitis) in the analysis
